# Characterization of lncRNA-protein interactions associated with Prostate cancer and Androgen receptors by molecular docking simulations

**DOI:** 10.1016/j.bbrep.2025.101959

**Published:** 2025-03-09

**Authors:** Barkha Khilwani, Bhumandeep Kour, Nidhi Shukla, Sugunakar Vuree, Abdul S. Ansari, Nirmal K. Lohiya, Prashanth Suravajhala, Renuka Suravajhala

**Affiliations:** a Department of Zoology, University of Rajasthan, Jaipur, Rajasthan, India; b Department of Molecular Biology and Genetic Engineering, School of Bioengineering and Biosciences, Lovely Professional University, Jalandhar, Punjab, India; c School of Interdisciplinary Health Sciences, Central University of Rajasthan, Bandarsindri, Ajmer, Rajasthan, India; d The CA Prostate Consortium of India (CAPCI), Bioclues.org, Hyderabad, India; e Amrita School of Biotechnology, Amrita Vishwa Vidyapeetham, Clappana, 690525, Kerala, India; f Department of Biotechnology, Vignan's Foundation for Science, Technology & Research, Vadlamudi, Guntur, India

**Keywords:** Prostate cancer, Long non-coding RNAs, Androgen receptor, 5α-dihydrotestosterone (DHT), Differentially expressed genes, Molecular docking

## Abstract

Long non-coding RNA (lncRNAs) are known to be implicated in pathogenesis of a broad spectrum of malignancies. These are found to have a significant role as signal transduction mediators in cancer signaling pathways. Prostate Cancer (PCa) is emerging with increasing cases worldwide even as advanced approaches in clinical diagnosis and treatment of PCa are still challenging to address. To enhance patient stratification, there is an indefatigable need to understand risk that can allow new approaches of treatment based on prognosis. While PCa is known to have mediated androgen receptor (AR) stimulation, the latter plays a critical role in regulating transcription of genes via nuclear translocation which in turn leads to response to androgens. LncRNAs have been implicated in developing clinical diagnostic and prognostic biomarkers in a broad spectrum of cancers. In our present study, 12 lncRNAs identified from clinical samples from our erstwhile PCa patients were docked with PCa and AR targeted 36 proteins. We identified three lncRNAs, *viz.* SCARNA10, NPBWR1, ANKRD20A9P are common between the targeted proteins and discern that SCARNA10 lncRNA could serve as a prognostic signature for PCa and AR biogenesis. We also sought to check the coding potential of interfacial residues associated with lncRNA docking sites.

## Highlights

• Potential for identifying prognostic signatures in PCa detection remains a focus.
• Identification of lncRNAs among DEGs is crucial for understanding PCa and AR regulatory mechanisms.
• SCARNA10 was identified as a common lncRNA in both PCa and AR, suggesting insights into PCa progression.
• Modulating lncRNA activity presents a novel approach for treating AR-related diseases.
• Understanding lncRNA interactions with AR could lead to advanced diagnostic and personalized medicine strategies.

## 1 Introduction

Prostate cancer (PCa) ranks as the second most prevalent type of cancer among men, with estimations suggesting that global cases will reach around 1,017,712 by the year 2040 [1,2]. With an increasing number of cases, multiple management options have been developed for diagnosis and treatment of PCa. These advances have enabled patient stratification by risk measurement and allow devising strategy of treatment based on prognosis and patient preferences [3]. PCa is dependent on androgen stimulation mediated by the androgen receptor (AR) even as AR plays a crucial part in the development and differentiation of the healthy prostate gland [4]. For example, it is well known that the AR complexes move to the nucleus and dimerizes before modulating the transcription of targeted genes wherein many genes become regulatory and invite candidate genes to interact with. Furthermore, it also allows the drugs to be targeted on their AR grooves, thus making the active functional motifs.

The gene AR, situated on the chromosome X at the Xq11-12 position and comprises of eight exons that encodes an open reading frame of about 2757 base pairs, resulting in a 10. kb kb mRNA having 919 amino acids (AAs) in it [5]. Its genetic structure remained preserved across mammalian species. The AR protein features several functional domains, including the amino-terminal domain (NTD), which comprises 555 AAs from exon 1; the DNA-binding domain (DBD, composed of 68 AAs from exons 2 and 3; and the ligand-binding domain (LBD), which contains 295AAa from exons 4 to 8. The nuclear localization signal is found between AA 628 and 657. The three transactivation units AF-1, AF-2 and Af-5 are encoded by exons 1 and 8. LBD and DBD are separated by a hinge region. The LBD, N-terminal and DBD hinge segments are highly conserved, however, NTD is more variable, featuring repetitive DNA sequences like GGC and CAG repeats. The DBD of AR, like any other nuclear receptors, includes 9 cysteines, among them 8 binds with 2 zinc ions, forming 2 zinc finger domains through sulfhydryl groups. Over time, extensive RNA profiling studies in PCa has led to the discovery of numerous lncRNAs that are dysregulated. Among those, 121 intergenic non-coding RNA(ncRNAs) associated PCa, known as the PCAT family, have been identified [6]. These lncRNAs significantly influence PCa by targeting critical pathways and regulatory mechanisms, such as chromatin remodeling, AR signaling, and PTEn/Akt. The AR, itself a member of the steroid and nuclear receptor superfamily, specifically the NR3C4 subgroup, plays a key role in growth and development of several organs [7]. As ligand dependent transcription factor, the AR is activated by binding to androgens such as testosterone and the 5α-dihydrotestosterone (DHT), which initiates sexual development and the differentiation of males. Essentially, AR responds to androgen binding by controlling gene transcription through its movement into the nucleus [7].

With the advancement of high-throughput sequencing technologies like next-generation sequencing (NGS) has greatly expanded our knowledge of different diseased characteristics, cancer being one of them. Our whole exome sequencing (WES) approach in PCa has identified ca. 30 causal genes in the Indian sub-population [2]. Similarly, the RNA-sequencing (RNA-seq) approach is used to measure the expression across a transcriptome and identify gene fusions, single nucleotide variants etc. In a parallel study in our lab, using the RNA-seq approach,we have earlier identified many lncRNAs and differentially expressed genes (DEGs) in PCa samples [8]. LncRNAs are recognized potential biomarkers for both prognosis and diagnosis in different cancers [9] with well-known lncRNAs such as XIST, HOTAIR, and MALAT are identified in genitourinary cancers but their role as biomarkers is warranted [10]. From our study, we identified ca.12 lncRNAs, few of them are known to be associated in several types of cancers.

The lncRNAs have crucial function in the epigenetic regulation as nuclei primarily aid in gene transcriptional phase resulting in changes in DNA, histone, acetylation and methylation. We have earlier shown how lncRNAs have a significant impact on pathogenesis of various malignancies attributing to their role as vital mediators in cancer-related signaling pathways [11]. Association of lncRNAs with metabolic intermediates, proteins, signaling molecules, cellular lipids etc., facilitates intracellular signaling with an aid of lncRNAs and their target molecules correlating to tumor progression. Important role of lncRNAs has been identified in development of PCa, promotion of castration resistant PCa (CRPC), cell proliferation, invasion, metastatic spread along with modulation of AR-mediated signaling [12]. One of the first lncRNAs to be described in PCa was prostate cancer gene expression marker 1 (CGEM1), a lncRNA that inhibits apoptosis and promotes cell proliferation *in vitro* via enhanced androgen-dependent gene transcription [13]. Another lncRNA, LINC01126 was identified as an AR-repressed lncRNA which was later found to be upregulated in CRPC. LINC01126 stabilized AR protein by enhancing AR nuclear translocation and its transactivation [14].

On the other hand, AR signaling pathway involves various mediators regulated by lncRNA using various mechanisms, for example, Prostate cancer antigen 3 (PCA3) modulates PCa cell survival via modulating the AR signaling and is now used in PCa diagnosis [15], SChLAP1 (second chromosome locus associated with prostate-1) was identified as a highly prognostic lncRNA that differentially expressed in aggressive and indolent form of PCa [16]. Several other lncRNAs, including PCAT1, PCAT19, PCA3, and PCGEM1, have also been reported to play important biological roles in PCa by regulating prostate cancer cell proliferation, metastasis and also by modulating AR expression. Recent studies have highlighted the role of many novel lncRNAs in PCa development and progression. Shi et al. have identified PCLN16, a novel lncRNA which augments AR signaling by interacting with Huntingtin interacting protein 1 (HIP1) [17]. Zhu et al. also reported the lncRNA activator role of STAT3 in PCa. STAT3 induced the expression of LINC00160 bound to EZH2 leading to the hypermethylation of RCAN1 and promoted the proliferation and metastasis of PCa cells [18].

Considering the dynamic role of lncRNAs as novel prognostic, diagnostic and predictive markers in PCa, they could also be potential targets for therapy, aiding in prevention, progression and treatment of CRPC and metastasis of the disease. Functional analysis of lncRNAs could be done by deciphering lncRNA-protein interaction as the function of most lncRNAs is dependent on interaction with protein-coding genes.

Despite the advances, there remain critical gaps in our understanding of the functional mechanisms underlying lncRNA involvement in PCa, particularly with respect to their interaction with proteins such as AR. This is an area where computational tools can significantly contribute, allowing for the prediction and characterization of lncRNA-protein interactions that are difficult to study experimentally. By identifying lncRNAs that interact with AR and contribute to PCa progression, we can potentially identify new prognostic biomarkers and therapeutic targets. However, to move beyond the computational predictions, it is essential to further explore the functional roles of these lncRNAs through experimental validation, including *in vitro* and *in vivo* studies.

This study aims to explore the mechanisms of lncRNA biogenesis and their interactions with AR-associated proteins in PCa, providing insights that could inform future research and clinical applications. By focusing on lncRNA-protein interactions, this work will contribute to the growing body of knowledge on how lncRNAs regulate AR signaling in PCa, with the potential to uncover novel biomarkers for diagnosis, prognosis, and therapy, particularly for CRPC and metastatic disease.

## 2 Materials and methods

### 2.1 PCa associated proteins, LncRNAs and androgen receptors (AR)

**Fig. 1:**
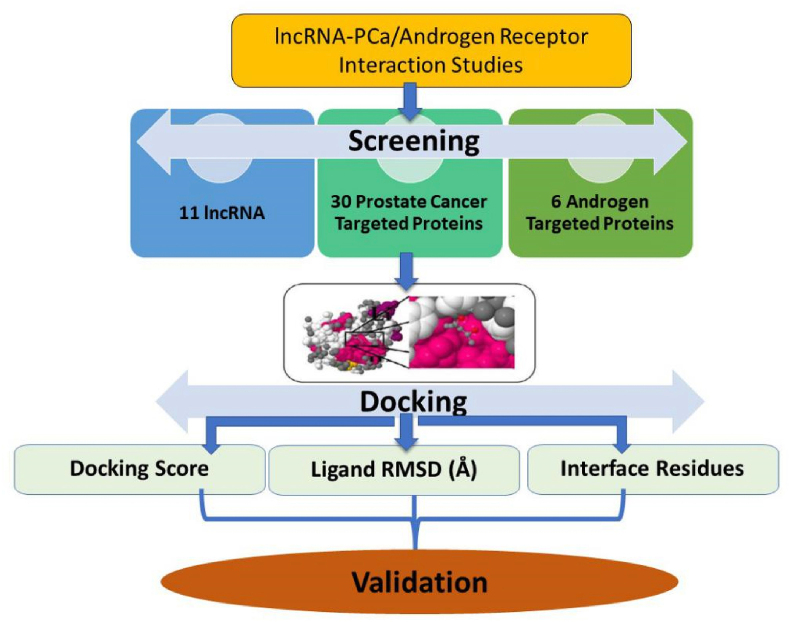
Systematic workflow of the 11 lncRNAs and the 36 targeted PCa and AR Proteins.

FASTA files of all the 28 PCa associated proteins, *viz. ADA, ANG, BRCA1, CTNS, HBB, GNPTAB, COL6A1, OTOF, TP53, CYP11B2, CYP1B1, GJB6, RHAG, DNAAF1, BRCA2, NF1 MCM8, MCCC1, CAPN3, MYO15A, MRE11, KRIT1, HEXB, SCN9A, PRLR, OPA1, ATP6V0A2 and USH2A* (Table 1) were retrieved from the NCBI (www.ncbi.nlm.nih.gov last accessed on May 25, 2024). The sequence data of 11 lncRNAs (SCARNA10, LINC01973, LINC00940, NPBWR1, FLJ16779, ANKRD20A9 P, LINC00298, SNHG19, LOC341056, TLX1NB, LINC00662:60) were extracted from NONCODE (http://www.noncode.org/last accessed on May 25, 2024), LNCipedia (https://lncipedia.org/last accessed on May 25, 2024) and RNAcentral (https://rnacentral.org/last accessed on May 25, 2024) to get their HSAT ids’ and their respective FASTA files. The proteins were considered as receptors and LncRNAs as ligands with the FASTA files rendered as an input to the HDOCK server (Fig. 1).

**Table 1:** List of PCa Proteins and PDB Ids used in the molecular docking study.

PCa Proteins		
PROTEIN	PDB ID	REFERENCES
**ADA**	3IAR	[19]
**ANG**	4AOH	[20]
**ATP6V0A2**	3RRK	[21]
**BRCA1**	6GVW	[22]
**BRCA2**	1MIU	[23]
**CAPN3**	6BGP	[24]
**COL6A1**	1KNT	[25]
**CTNS**	7ZKW	[26]
**CYP11B2**	7M8I	[27]
**CYP1B1**	6OYV	[28]
**GJB6**	5ER7	[29]
**GNPTAB**	2N6D	[30]
**HBB**	6LCX	[31]
**HEXB**	3LMY	[32]
**KRIT1**	5D68	[33]
**MCCC1**	2EJM	[34]
**MCM8**	6L0O	[35]
**MRE11**	3T1I	[36]
**MYO15A**	7UDU	[37]
**NF1**	1NF1	[38]
**OPA1**	6JTG	[39]
**OTOF**	3L9B	[40]
**PRLR**	3NPZ	[41]
**RHAG**	8CSX	[42]
**SCN9A**	7W9M	[43]
**TP53**	6VTC	[44]
**Androgen Receptors**		
**Androgen Receptor (AR)**	4QL8	[45]
**Androgen receptor (AR)**	2AM9	[46]
**Androgen receptor (AR)**	2PNU	[47]
**Androgen receptor (AR)**	1E3G	[48]
**Selective Androgen Receptor Modulator (SARM)**	5V8Q	[49]
**Selective Androgen Receptor Modulator (SARM)**	5CJ6	[50]
**Uroporphyrinogen Decarboxylase (URO-D)**	2Q71	[51]

To further narrow down lncRNAs, we checked whether or not any of these lncRNAs show any interaction with 30 causal genes obtained from our WES study. We used LncPro (http://cmbi.bjmu.edu.cn/lncpro last accessed on May 21, 2024) and RPI-Pred (http://ctsb.is.wfubmc.edu/projects/rpi-pred last accessed on May 21, 2024) for predicting the interaction between lncRNAs and protein-coding genes (supplementary files) and finally chose 5 lncRNAs which were showing strong interaction (above 90 %) with some of the very important causal genes of PCa. For ARs (2Q71, 5V8Q, 4QL8, 2PNU, 5CJ6, 2AM9, 1E3G, 7KW7) we gave their protein data bank (PDB) id and chain ids as input. Both PDB and chain id were taken from RSCB PDB (https://www.rcsb.org/t accessed on May 25, 2024). All the ARs were then considered as receptors and lncRNAs as ligands.

### 2.2 Molecular docking studies

Docking analysis was performed using HDOCK (http://hdock.phys.hust.edu.cn/last accessed on May 25, 2024), a docking tool. Numerous biological activities, including signal transmission, cell control, protein synthesis, DNA replication and repair, RNA transcription, etc., depend on interactions between nucleic acids and proteins. Thus, understanding their intricate structure will help researchers design treatment strategies or medications that specifically target these interactions. Because experimental approaches are expensive and technically challenging, molecular docking has become crucial in the identification of complex structures [52]. For protein-protein docking, the HDOCK server (http://hdock.phys.hust.edu.cn last accessed on May 1, 2024) combines homology search, template-based modeling, structure prediction, macromolecular docking, biological information incorporation, and task administration. The server automatically predicts the interaction between receptor and ligand molecules using input data for both molecules (amino acid sequences or PDB structures). This was done using a hybrid method of template-based and template-free docking [53].

### 2.3 Workflow of molecular docking

1. **Input:** Both protein sequences and structures were accepted as input data in the workflow's initial step. The HDOCK server was built to take inputs for both protein sequences and structures, which makes it easier for both inexperienced and regular users to operate. The server takes two types of inputs for structures and two types of inputs for sequences for every molecule, given: 1) A PDB-formatted pdb file. ii) pdb file with chainID in PDB (e.g. 1CGI:E). iii) copy the protein sequence and paste it in the FASTA format. iv) Uploading a FASTA-formatted protein sequence file. Each molecule just requires one kind of input and with automated modeling of DNA/RNA structures from sequences currently difficult, the service only accepts structure inputs for DNAs and RNAs at this time [52].
2. **Sequencing similarity:** To determine the homologous sequences for both receptor and ligand molecules, this similarity match was carried out against the PDB sequence database using the sequences from input or converted from structures. The HHSuite software was used for protein sequence searches since it is widely known because of its effectiveness in locating distant homologs. Since FASTA (version 3.6) is a powerful and user-friendly tool for both protein and DNA/RNA sequence search, it is utilized for DNA/RNA and thus two sets of homologous templates are produced as a result of this process.
3. **Template selection:** After that, the process moves on to the third stage, which involves comparing two sets of templates to check if they shared any entries having the similar PDB codes. A similar template was chosen both for the receptor and the ligand if there are any such PDB codes. The best templates for the receptor protein and/or ligand protein were chosen from two sets of homologous templates, respectively, assuming that there was no link between the two sets. When there are many templates present, then the one with maximum sequence coverage, sequence similarity, and resolution is chosen. Models are constructed using MODELLER with the chosen templates, and ClustalW was used for sequence alignment.
4. **Result:** As the HDOCK server adds docking tasks to the queue on providing input three task status including “QUEUED,” “RUNNING,” and “RESULTS,” are yielded and finally the docking results found at http://hdock.phys.hust.edu.cn/date/jobid, where “jobid” is the specific job id displayed on the web page of status was retrieved.
5. **Output:** The docking output consists of three fundamental files: Receptor PDB file created by the server using the users' FASTA sequence or supplied by users, Ligand PDB file created by the server using the user-provided FASTA sequence or supplied by users and ligand binding modes reflected by their transformations in the HDOCK output. Additionally, the result page displays a docking summary of the top 10 models at the bottom and the template information for the receptor and ligand at the top [52].

Based on the highest ligand receptor binding energy, an interacting model of each interaction was selected and these models were then subjected to visualization.

**Visualization:** The docked scores of lncRNA with proteins generated 180 complexes with 10 best poses for each lncRNA-protein resulting in 1800 models. Screening of the models was carried by least-bind energy complexes; 12 models of the complexes were considered to be identified of which, 5 complexes with PCa and 7 complexes with AR. The lowest energy known to have more stability was considered for 3D visualization with PyMOL software. The parameters were assigned to ligand site hydrogen bonds with <3 Bond distances were identified that indicate the high intensity and the possible orientation of the proteins resulting in the stable complex formation.

**Coding potential:** To check if there is any coding potential attributing to the interfacial residues, we performed checks using intrinsic feature estimation by employing CPC2 [54]. The tool works on the premise that the ORF length coverage is estimated along with Fickett and Hexamer scores which judiciously classify estimating ncRNAs. The Fickett score is based on frequencies of A, T, G, and Cs leaning upon the intrinsic divergence between ncRNAs.

## 3 Results and discussion

From our recently published RNA-Seq analyses [8], 11 lncRNAs identified (SCARNA10, LINC01973, LINC00940, NPBWR1, FLJ16779, ANKRD20A9 P, LINC00298, SNHG19, LOC341056, TLX1NB, LINC00662:60*)* were screened in the form of FASTA files which were then retrieved as discussed previously (using NCBI, NONCODE, LINCipedia and RNA central public portals). We considered the HDOCK web server for molecular docking studies to identify the interactions of receptor-ligands. Among the 11 lncRNAs (Refer materials), 6 lncRNAs LINC01973, FLJ16779, LINC00298, SNHG19, LOC341056, Land INC00662:60 with a limit of 5000 residues were not done as the interactions were not deciphered with no alternate tools available to consider. We performed docking on 5 lncRNAs (SCARNA10, LINC00940, NPBWR1, ANKRD20A9 P, and TLX1NB) with 28 PCa and AR-targeted proteins. Five lncRNAs (SCARNA10, LINC00940, NPBWR1, ANKRD20A9 P, and TLX1NB) with 27 PCa-associated proteins except USH2A resulted best confirmers each generated 10 models of which *CTNS (PDB ID:* 5CTG*); ANG(PDB ID:* 4AOH*); CYP1B1 (PDB ID:*3PM0*)* observed to show more stable complex formation (Table 2).

**Table 2:** The potential candidate 5-lncRNAs’ interactions with 3-PCa proteins (PDB id) and binding energies, root mean square deviation (RMSD), and Interacting residues.

S.No.	LncRNA	PCa Protein	PDB ID	Docking Score	Ligand RMSD (Å)	Interface Residues
1	TLXINB	CTNS	7ZKW	−322.82	57.44	Ser177, Lys 176, Trp 304, Asn 322, Asp 324, Phe 317, Thr 261
2	SCARNA10	ANG	4AOH	−259.38	482.89	Gly72, Asn87, Lys89, Ser99, Lys 97, Phy100
3	NPBWR1	ANG	4AOH	−230.33	740.84	Lys41, Gly72, Ser99, Arg146
4	ANKRD20A9 P	ANG	4AOH	−220.15	803.24	Asn 73, Ser 99, Lys 97
5	LINC00940	CYP1B1	6OYB	−201.96	3095.39	Gly72, Ser99, Ile143

### 3.1 LncRNA-PCa interactions

**Fig. 2:**
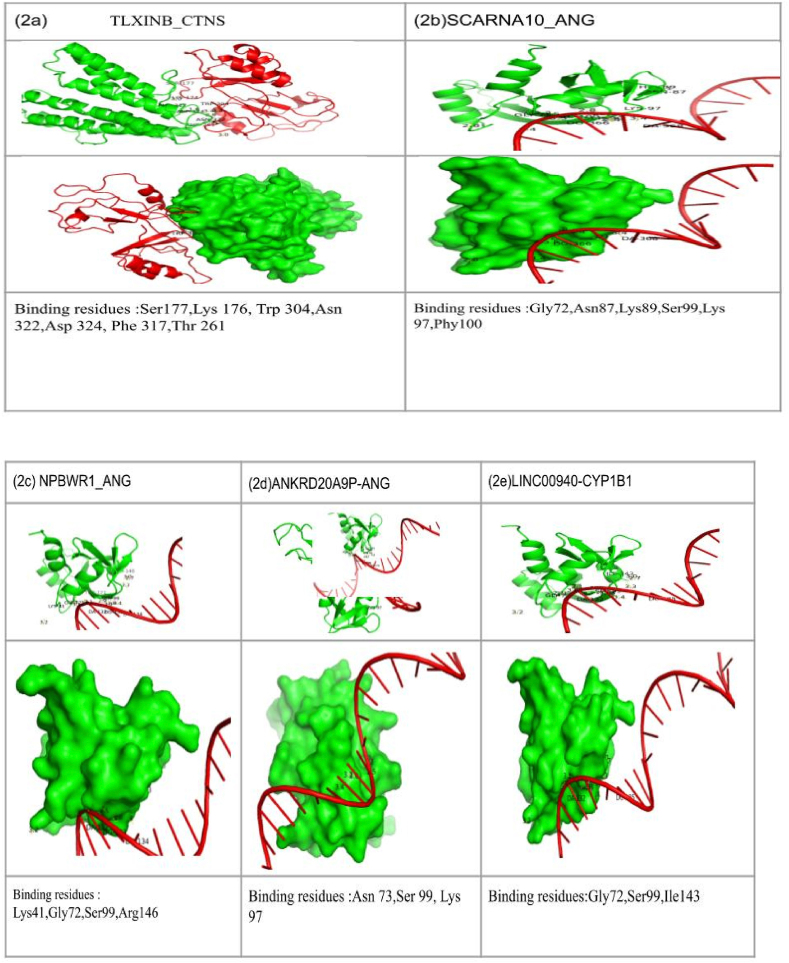
The 3D visualization using Pymol the complexes of potential 5-lncRNAs and interacting residues with 3-PCa proteins (2a-2e).

The docked complexes of TLXINB-CTNS (PDB ID: 5CTG) with binding energy −322.82 kcal/mol and interacting/interfacial residues were identified, *viz.* Ser177, Lys 176, Trp 304, Asn 322, Asp 324, Phe 317, Thr 261 (Fig. 2a); SCARNA10 -ANG(PDB ID: 4AOH) observed to have the least binding energy −259.38 kcal/mol and interacting residues was identified as Gly72, Asn87, Lys89, Ser99, Lys 9, Phy100 (Fig. 2b); in complex NPBWR1-ANG(PDB ID:4AOH) −230.33 kcal/mol and interacting residues was identified as Lys41, Gly72, Ser99, Arg146 (Fig. 2c); in complex ANKRD20A9 P-ANG (PDB ID: 4AOH)-220.15 kcal/mol and interacting residues was identified as Asn 73, Ser 99, Lys 97 (Fig. 2d), while the LINC00940-CYP1B1 (PDB ID:3PM0) −201.96 kcal/mol and interacting residues were identified as Gly72, Ser99, Ile143 (Fig. 2e).

### 3.2 Validating LncRNA-AR interactions

**Fig. 3:**
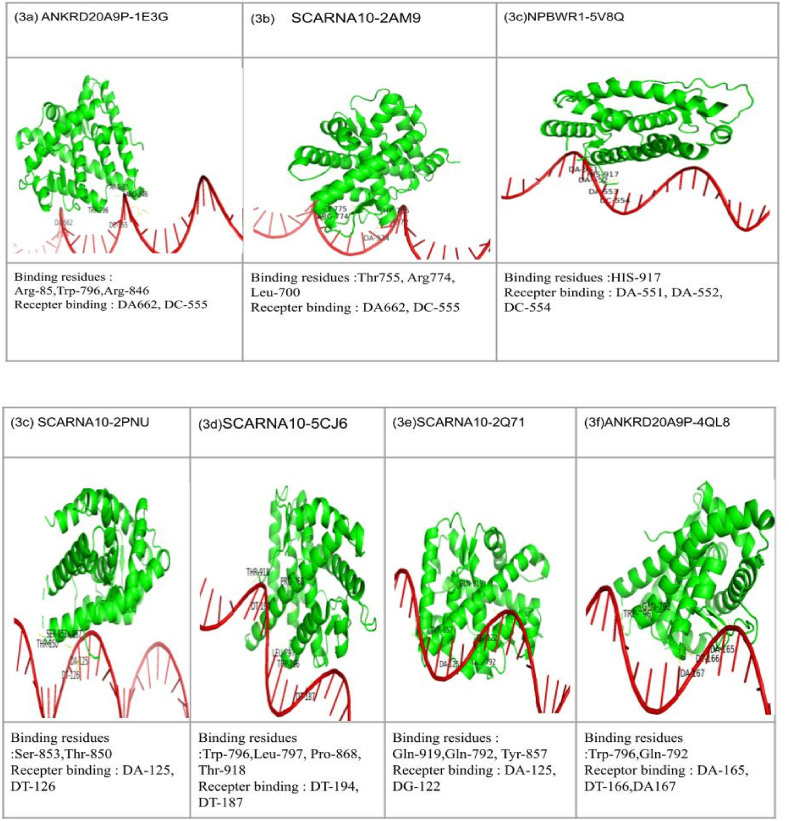
The 3D visualization using Pymol of for the complexes of potential 5-lncRNAs and interacting residues with 7 targeted AR proteins (3a-3f).

**Fig. 4:**
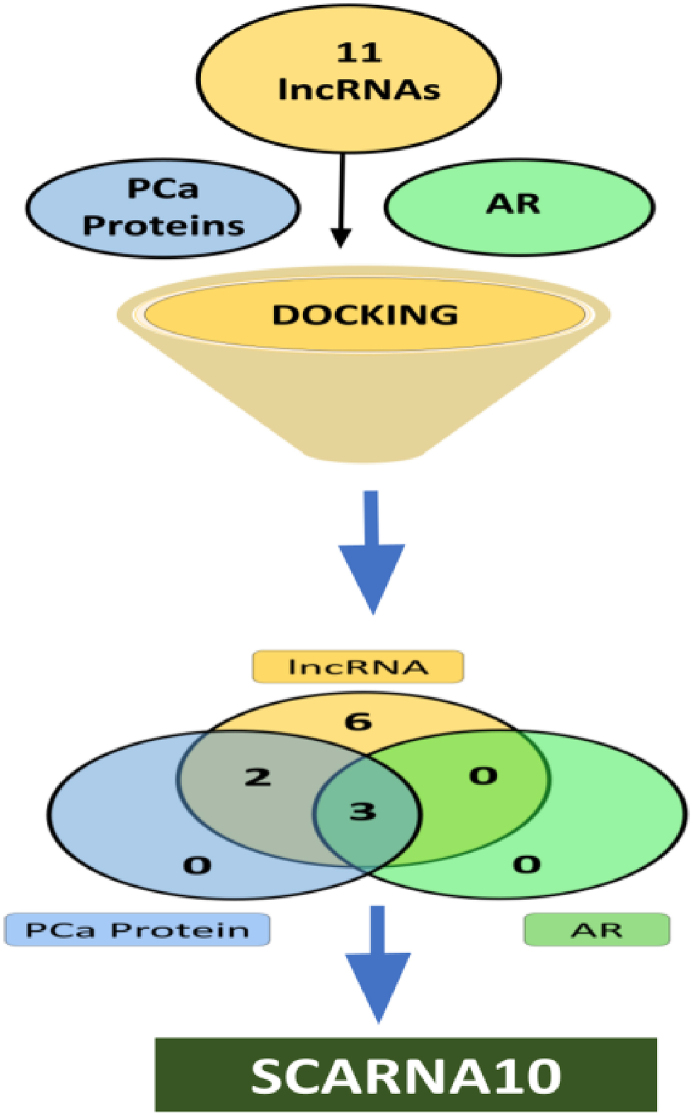
Summary of scoring results obtained after docking of 11 lncRNAs with PCa-associated proteins and AR following identification of SCARNA10 (lncRNA) as a common potential target.

**Fig. 5:**
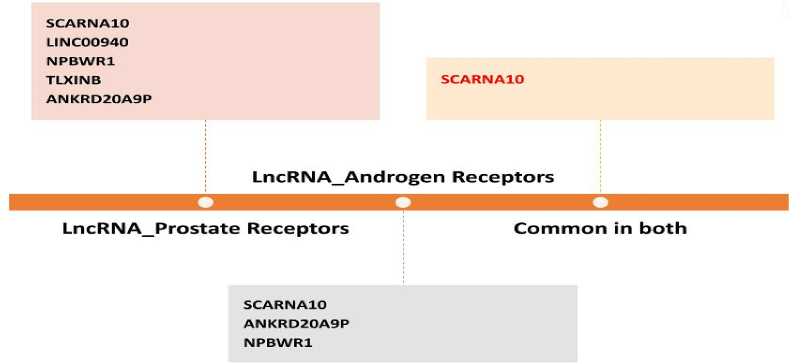
Overview of 11 lncRNAs interactions with 28 PCa proteins and 7 protein studies to identify potential lead SCARNA10 lncRNA that is common.

SCARNA10, LINC00940, NPBWR1, ANKRD20A9 P, TLX1NB were considered to know the possible interaction with AR targeted proteins (PDB id:2Q71, 5V8Q, 4QL8, 2PNU, 5CJ6, 2AM9, 1E3G, 7KW7) were considered. From the interaction studies, we identified least binding energy in 1E3G-ANKRD20A9 P, 2AM9-SCARNA10, 5V8Q-NPBWR1 with -212–208 kcal/mol binding energy as shown in Table 3. 1E3G-ANKRD20A9 P has binding energy of −212.74 kcal/mol, with ligand RMSD of 742.99 Å and residues are Arg-85,Trp-796,Arg-846 (Fig. 3a); 2AM9-SCARNA10 with binding energy of −208.93 kcal/mol, ligand RMSD as 536.64 Å and the interaction residues are Thr755, Arg774, Leu-700 as shown in Fig. 3(3 b); While 5V8Q-NPBWR1 with −208.04 kcal/mol, ligand RMSD 677.49 Å of and interacting residues was identified as His-917(Fig. 3c); 2PNU-SCARNA10 with binding energy as −201.39 kcal/mol, RMSD 613.81 Å and interacting residues Thr-850, Ser-853,Tyr-857 (Fig. 3d); 5CJ6-SCARNA10 complex has binding energy of −197.78 kcal/mol, RMSD of 631.85 Å and binding residues are Trp-796,Leu-797,Pro-868, Thr-918 (Fig. 3e; and 2Q71-SCARNA10 complex showed binding energy of −193.91 kcal/mol, RMSD of 634.84 Å and its binding residues are Gln-919,Gln-792, Tyr-857(Fig. 3f) were visualized the 3D complexes with hydrogen interactions bellow 3 Å bond distance. While in 4QL8-ANKRD20A9 P, we obtained the highest binding energy of −185.99 kcal/mol with 762.89+ Å as ligand RMSD value. Our study indicates the SCARNA10 as a potential lncRNA having the lowest binding affinity energy of −259.38 with SCARNA10-ANG and −259.38 with SCARNA10-2AM9) suggesting their potency (Fig. 4, Fig. 5).

**Table 3:** The potential candidate 5-lncRNA interactions with 7-AR proteins (PDB id) and binding energies, RMSD and interacting residues.

S.No.	LncRNA	Androgen Receptor Protein	Docking Score	Ligand RMSD (Å)	Interface Residues
1	**ANKRD20A9P**	**1E3G**	**−212.74**	**742.99**	**Arg-85,Trp-796,Arg-846**
2	**SCARNA10**	**2AM9**	**−208.93**	**536.64**	**Thr755, Arg774, Leu-700**
3	**NPBWR1**	**5V8Q**	**−208.04**	**677.49**	**His-917**
4	SCARNA10	2PNU	−201.39	613.81	Thr-850, Ser-853,Tyr-857
5	SCARNA10	5CJ6	−197.78	631.85	Trp-796,Leu-797,Pro-868, Thr-918
6	SCARNA10	2Q71	−193.91	634.84	Gln-919,Gln-792, Tyr-857
7	ANKRD20A9 P	4QL8	−185.99	762.89	Trp-796,Gln-792

Recent studies have revealed that lncRNAs can regulate androgen signaling through various mechanisms [55]. It has been reported that lncRNAs can transactivate AR by binding to its enhancer region. LncRNAs are significant in prostate tumorigenesis as gene transcription regulatory sequences. PCGEM1, HOX transcript antisense RNA (HOTAIR), and PCa gene 3 (PCA3) are some examples of lncRNAs that function as oncogenic and/or tumor suppressors in PCa through the AR signaling pathway [56] (Zang et al., 2016). Deficiency effect was reported in androgen-related transcription factor 1 (GDS5606/7953383) and carboxyl terminal-binding protein 2 in (FoxA1 Knockdown) studies, Peptidyl-prolyl cis/trans isomerase Pin 1 (GDS5805/7953383) LNCaP. The phased mutation and SV Integrated studies reported that in LAPC4/LAPC4-CR cell lines duplications were found to increase due to CDK12 mutations [44]. While PC3 and VCaP cells were reported to have chromothripsis induced TP53 mutations, these reports indicate that high somatic mutations related to events in chromoplexy and chromothripsis have a critical role in PCa pathogenesis. A few limitations such as rearrangement were not considered in aneuploidy. In our lab, RNA-seq data from a small cohort of PCa patients was analyzed and some critical lncRNAs were identified as deterministic markers [8]. In the present study, docking analysis of prostate-specific proteins and AR with lncRNAs was performed to explore binding potential towards identifying interacting residues and putative RNA-binding motifs. Androgen signaling plays a vital role in PCa development and in treatment strategies [57] as AR splice variants, amplification/overexpression of androgens, and AR-Ligand binding stimulate the development of CRPC. Our studies employed identifying target regions in AR to modulate and control AR-signaling pathways. Androgen-dependent signaling instigates the progression of PCa from benign to malignant disease with molecules like enzalutamide and abiraterone acetate playing a critical role as second-generation anti-androgen therapy [58].

From the docking studies, SCARNA10, a candidate lncRNA common to both the PCa and AR targeted proteins was identified as it is known to inhibit targeted gene binding of a promoter to polycomb repressive complex 2 (PRC2) suppressing the TGF signaling, we aimed to understand the mechanism of SCARNA10, when silenced, reducing the levels of TGF, TGF R1, KLF6 and Smad 2,3 [59,60]. It was also reported that SCARNA10 has a major role in chromosomal mutation at 12p13.31 identified in PCa metastasis studies [61]. In a few studies, SCARNA10 was reported to be up-regulated in breast and lung cancer [62]. Very limited data was reported of which the NCBI/GEO datasets reported that SCARNA 10 in PCa considering LNCaP cell line. Furthermore, it was identified that mR-135 b, FOXA1 (GDS4957/ILMN_3246209; LNCaP.FoxA1.6) were found to be overexpressed in clinical human PCa samples, VprBP depletion effect (GDS4829/ILMN_3246209) was reported. The studies provide potential lncRNA prognostic biomarkers for future PCa research.

### 3.3 Coding the uncoded

We observed that NONHSAT032215.2 is a human non-protein coding lnc-TUBA3C-16:1 gene and NONHSAT126578.2 have complete ORFs with putative peptides while NONHSAT026096.2 is with an incomplete peptide indicating even as the probability of coding potential is par 5 %. We argue that these regions could be at the interfacial sites and serve as candidate regions for selective epitope binding (Table 4).

**Table 4:** Coding potential of lncRNAs along with their putative peptides.

S.No.	LncRNA	Nucleotide sequence with soft masking in small letters	Coding putative peptide
1	NONHSAT032215.2	CATGAAGAAAGACGTGCGGATCCTGCTGGTAGGAGAACCTAGAGTTGGGAAGACGTCACTGATTATGTCTGGTCAGTGAAGAATTTCCAGAAGAGGTTCCTCCCCGGGCAGAAGAAATCACCATTCCAGCTGATGTCACCCCAGAGAGAGTTCCAACACACATTATAGATTACTCAGAAGCAGAACAGAGTGATGAACAACTTCATCAAGAAATATCTCAGGCTAATGTCGTCTGTATAGTGTATGCCGTTAACAACAAGCATTCTATTGATAAGGTAACAAGTCGATGGATTCCTCTCATAAATGAAAGAACAGACAAAGACAGcaggct	MKKDVRILLVGEPRVGKTSLIMSGQ
2	NONHSAT126578.2	TAAACTAATGCAATAATTCAATGAAGATATGGTAATAGAGGTACAATAGAAGTTCAAAATAAATAAGAAACATGTTAGACAGGTTTGTGAAATTCTTCAACTATGGTATCTGATAGGAGTGATGCTGAACAAGGCAGATGTTACCGACTAGATGTTAAAAGAAGATTAATTAAAATGAGAAAATGTCTTAGACCAGAAAAACTGATGAATTCGATGAACCAAAGAGAAATAAGCATGCAACATGAACAGCCAGAAGAAAGTTTTCAGGAACTAGTGGAAGATTACTGATGTGTTATTGAAGGACTCATTCAAGAGTAA	MVSDRSDAEQGRCYRLDVKRRLIKMRKCLRPEKLMNSMNQREISMQHEQPEESFQELVEDY
3	NONHSAT026096.2	GGACCTTTGGCCTGTTAAAGGTCTGTAATCTTGGTGGGCGATACAGAGTTATGTGTGTTCACTGTAAGGGCAGACCAACAAGAACTTTTTCCTACTTTTGAGCTACCTCTTTTTAATAGGGGTGATTCTTCCAGTTGCTGGAGAGAAATTGTGGTAACTGGAGTGAGAGAGTAGGAACAGGGCATGTTCAGGGTATCAGGGCCAAGGGTCCTAAAGGACTTAGCTTGTGTTATGGCCACTGAGAGATG	MFRVSGPRVLKDLACVMATER

What remains intriguing is the coding potential of lncRNAs as we have considered those potential coding peptides and looked for similarity searches. For example, NONHSAT032215.2 was found to have similarity to mitochondrial Rho GTPase 1 isoform and RHOT genes and these potential peptides were indeed suppressed over the evolution. Deregulated Rho GTPases have been discovered in various tumors, including the prostate, which are known to promote the metastatic properties of human cancer cells [63]. Similarly, Histone acetyltransferase 1 is known to upregulate AR expression to modulate CRPC cell resistance to well-known drugs such as enzalutamide [64] and recently it is shown to epigenetically influence the AR signaling [65].

## 4 Conclusions

With the advent of NGS approaches, identifying lncRNAs among the DEGs play a crucial role to bridge the gap between the regulatory mechanisms of PCa and AR. In this work, we attempted to dock lncRNAs discovered from our previous PCa analysis to that of AR and infer candidate interactions. While we identified SCARNA10 as common lncRNA between both PCa and AR and argue that the functional aspect of this lncRNA would give us some insights into PCa progression, there is a room for identifying putative prognostic signatures for PCa detection. We also sought to ask whether or not any interfacial residues are at the site of coding potential of lncRNAs. As a future perspective, we envisage identifying specific lncRNAs that play crucial roles in AR signaling which could lead to the development of targeted therapies and further perform *in vitro* validation. Modulating the activity of these lncRNAs could potentially be a novel approach for treating AR-related diseases. Furthermore, understanding their interactions with AR could lead to the development of diagnostic or prognostic tools. If specific lncRNAs are found to be critically involved in AR signaling, we believe that this knowledge could be applied in the context of personalized medicine. Tailoring treatments based on a patient's unique genetic and molecular profile could lead to more effective therapeutic strategies. Nonetheless, there could be microRNAs as well (miRs) at the helm of these interfacial sites, but as lncRNAs are largely regulatory, we deem to bridge the gap between lncRNAs and proteins associated with AR signaling.

## Funding

None.

## CRediT authorship contribution statement

Barkha Khilwani,Renuka Suravajhala, Prashanth Suravajhala: Conceptualization, Barkha Khilwani, Bhumandeep Kour, Nidhi Shukla, Renuka Suravajhala: Methodology,Visualization, Writing- Original draft preparation, Software Abdul Ansari, Nirmal K Lohiya, Sugunakar Vure, Prashanth Suravajhala: Data curation, Investigation. Renuka Suravajhala: Supervision. Prashanth Suravajhala, Renuka Suravajhala and Nirmal K Lohiya Writing- Reviewing and Editing

## Declaration of competing interest

The authors declare that they have no known competing financial interests or personal relationships that could have appeared to influence the work reported in this paper.

## Data Availability

The data that has been used is confidential.
